# Resident Exposure to Acutely Ill Patients Over the Course of Residency

**DOI:** 10.5811/westjem.63704

**Published:** 2026-09-24

**Authors:** Daniel J Hekman, Joe-Ann Moser, Benjamin H Schnapp

**Affiliations:** * University of Wisconsin-Madison, BerbeeWalsh Department of Emergency Medicine, Madison, Wisconsin; † University of Wisconsin-Madison, Department of Population Health Sciences, Madison, Wisconsin

## Abstract

**Introduction:**

Emergency medicine literature has long debated the “black cloud” and “white cloud” phenomena, which suggest individual variability in exposure to acutely ill patients. If true, this variation may have significant implications for resident training. This study investigates whether emergency medicine (EM) residents consistently encounter differing volumes of high-acuity clinical encounters throughout their training.

**Methods:**

This retrospective cohort study analyzed 11 years of electronic health record (HER) data from a Midwestern academic emergency department to quantify the number of acutely ill adult patients seen by EM residents across three years of training, using a consensus-based set of clinical criteria. Our primary outcome was the concordance of the ranking of each resident within their cohort by number of acutely ill patients seen during postgraduate year one (PGY 1) compared to PGY 3. Statistical analyses to assess this outcome included weighted kappa and linear regression.

**Results:**

A total of 93 EM residents met the criteria for inclusion and saw a total of 359,736 patients during their residency (average 3,493 per resident). Residents saw an average of 167.5 (19.8) acutely ill patients during their residency training. This volume increased sharply by year, with PGY-1 residents seeing an average of 23.8 (7.3) acutely ill patients, and PGY-3 residents seeing an average of 100.3 (19.4). Both weighted kappa (κ = −.274) and linear regression analysis (β = −0.84; 95% CI, −1.4 to −0.31; *P* = .002) indicated that a resident who saw more acutely ill patients than average in PGY 1 was more likely to see fewer acutely ill patients than average during PGY 3. Results were unaffected by the COVID-19 years or subsequent critical care fellowship pursuit.

**Conclusion:**

“Cloud type” (as measured by EHR-documented care for acutely ill patients) does not appear to be a stable trait over time. Our findings are consistent with previous studies that found no association between residents’ “cloudiness” and objective measures of workload. Using EHR data to track resident clinical exposures helps educators identify training gaps and target remediation through in situ or simulated experiences.

## INTRODUCTION

Emergency physicians have long been superstitious about the mysterious ebbs and flows of their practice environment, suspecting that everything from full moons,^1^,^2^ Friday the 13th,^3^ New York Yankees souvenir bat days,^4^ and use of the word “quiet”^5^,^6^ can impact emergency department (ED) volumes. Empirical studies of these phenomena have failed to find statistically or clinically significant, measurable differences in clinical volumes or patient acuity to back up these superstitions.

However, another medical superstition that may have greater empirical support is the concept of “black clouds” and “white clouds,” in which some physicians consistently see a higher or lower proportion of critically ill patients compared to their peers.^7^ A study by Zhao and colleagues found that “black cloud” hand surgeons saw a higher number of add-on cases and patient transfers.^8^ Similarly, Tan and colleagues found that “stormy” neurosurgery trainees saw more consults.^9^ However, empirical data supporting a “cloud type” for neurosurgery residents is not consistent across institutions,^10^ and a small study in a pediatric emergency medicine (EM) population found no signs of this effect when examining ED visits and admissions.^11^ Similarly, this effect has not been seen when studied in general surgery residents.^12^ More recently, researchers at a large health system in Turkey found that there was no statistically significant difference in daily volumes, admissions, intensive care unit (ICU) admissions, high-acuity triage patients, traumas, or in-ED deaths among clinicians deemed the “luckiest” or “unluckiest,” via a survey of the physician group.^13^ Importantly, the definition of “black cloud” varies across the published research, with some defining it based on patient volumes (eg, number of consultations) and others defining it based on patient acuity (eg, ED admissions).

If present, the acuity-based “black cloud” and “white cloud” phenomena would have important implications for clinical training. By the time of graduation, EM residents have been found to have significant differences in their exposure to a variety of patient types, including differences in overall patient complaint distributions,^14^ number of pediatric patients,^16^ and critically ill pediatric patients.^17^ Experiential learning theory suggests that clinical exposures are essential for growth by allowing residents to actively experiment with learned knowledge and critically reflect on what happened.^18^ “White cloud” residents with less clinical exposure to acutely ill patients than their cohort may lack these essential exposures and might benefit from additional opportunities to simulate these cases or have additional rotations in the ICU to bolster their experience. However, to our knowledge, no study has looked at variability in EM resident clinical exposure to acutely ill adult patients during their EM training.

The goal of this study was to determine whether some residents saw a significantly higher or lower number of acutely ill patients than other trainees. We also examined whether that difference was stable over time—that is, do residents have a stable cloud type—operationally defined as being in the top tertile (most acutely ill patients; “black cloud”) or bottom tertile (“white cloud”) within their residency cohort of electronic health record (EHR)-attributed exposure to acutely ill patients. We also assessed whether residents who pursue a critical care fellowship are more likely to have consistently treated acutely ill patients during residency training than their peers. The motivating goal of this work was to investigate the possibility of identifying residents at risk of having insufficient experience with treating acutely ill patients during residency and to provide residency leaders with a tool to track resident progress, allowing targeted intervention (eg, additional simulation lab time) as needed.

**Population Health Research Capsule**

What do we already know about this issue?

*The number of acutely ill patients a resident sees over residency is important for experiential learning and growth*.

What was the research question?

*Is exposure to acutely ill patients, relative to cohort, stable over residency?*

What was the major finding of the study?

*Residents saw an average of 167.5 (19.8) acutely ill patients during residency. We found a negative correlation between postgraduate year one (PGY-1) and PGY-3: (weighted Kappa −.274; 95% CI, −0.42 to −0.04)*.

How does this improve population health?

*Tracking resident exposures to acutely ill patients via the electronic health record can identify shortfalls and highlight need for increased exposure in situ or via simulation*.

## METHODS

### Setting

This retrospective cohort study used data from EM residents at an ED that treats approximately 70,000 patients per year. It is associated with an academic medical system and Level I trauma center in the Midwestern United States. The ED has both pediatric and adult beds with separate faculty, and residents work shifts in both areas. This program hosts a three-year residency program that currently accepts 13 residents per year (during 2015–2022 cohorts were 12 residents). We extracted 11 years of EHR data from July 2015 to June 2025, representing nine complete cohorts of EM residents. For most of the study period, the adult ED functioned under an “open” model where all patients (other than low-acuity patients seen in the fast track area) could be seen by any resident. For the years 2019 to 2022, the ED functioned under a pod system where an ED team was responsible for predefined beds. These models mostly affected faculty assignments because residents were allowed to assign themselves to patients when they felt ready to do so under both models.

All resident physicians who completed the residency program uninterrupted in the normally scheduled three years were included for analysis. The number of residents per cohort varied due to a small number of interruptions in or withdrawals from training, but most residency cohorts had 12 residents. We included all adult patient (greater than 18 years of age) encounters seen by any of the eligible residents; pediatric visits were excluded due to the relatively low overall percentage of acutely ill children and the potential need for different criteria to define acutely ill pediatric patients.

This was a retrospective review of EHR data, which was deemed exempt quality improvement by our institution’s health sciences institutional review board. Data were extracted—not abstracted—as discrete data from a relational database by an experienced data scientist (DJH); thus, abstractor-related measures outlined in Worster and Bledsoe were not applicable.^19^ SQL was used for data retrieval, and R was used for processing and analysis.^20^

### Primary Outcome

Our primary outcome was the number of acutely ill patients seen by each EM resident during each year of their residency. For the purposes of this study, we defined acutely ill based on the potential for acute decompensation while in the ED. We excluded categories such as “stroke code” activations and “code sepsis” activations because these activation criteria are intentionally broad and include patients who are not acutely ill. By contrast, patients receiving intravenous (IV) insulin drip were classified as acutely ill due to the use of medications exclusively for treating highly acute disease, including diabetic ketoacidosis and severe hyperkalemia. A patient was identified as acutely ill if they met any of the following criteria (documentation source):

- Emergency Severity Index (ESI) 1-Red (triage)
- Admission to ICU (admission, discharge, transfer [ADT] documentation)
- Level I trauma (triage)
- ST-elevation myocardial infarction (STEMI) (diagnosis)
- Death in ED (patient record)
- Central line placement (procedure order)
- Arterial line placement (procedure order)
- Vasopressors given (medication order based on pharmaceutical subclass)
- Vasodilators given (medication order based on pharmaceutical subclass)
- Anaphylactic shock (diagnosis)
- Ketamine IV drip (medication)
- IV insulin drip (medication)

This list was derived by consensus by a panel of experienced faculty emergency physicians. These criteria are dependent on institutional documentation practices and should not be interpreted as an exhaustive list of acutely ill criteria or a future “gold standard” applicable across all EDs. However, the authors and the residency leadership at our institution believe that, taken together, this list will likely identify nearly all acutely ill patients, especially those that residents at our program would be expected to gain experience treating per Accreditation Council for Graduate Medical Education guidelines. For example, a patient with severe respiratory distress requiring intubation or one requiring resuscitation is likely to arrive as an ESI 1 and/or be admitted to the ICU; therefore, the patient would be included in this analysis, even though intubation or resuscitation themselves are not included as a criteria due to difficulties within the data source of performing service attribution. Other studies and national benchmarks use billing information (eg, level of service 5 and/or critical care) to identify high-complexity patients;^21^ we chose not to use billing information because it relies on attending physician documentation rather than on clinical activities of the resident as documented in the EHR. While such a definition focused on resident documentation may have limitations, it was consistently applied to all residents across the study period to minimize bias.

### Resident Attribution

Resident physicians are matched with acutely ill patient care in three ways, depending on the type of acute illness flag. For medication-based flags, attribution was based on the resident who was the ordering clinician for the medication. For procedure-based flags, attribution was based on the resident who was the performing clinician listed on the procedure order in the EHR. For all other flags, resident attribution was based on the resident physician to first sign the primary clinician note in the ED. Thus, it was possible for the same patient to be attributed to multiple residents if the patient met multiple flags with different attributions. For example, for a patient who arrived as a Level I trauma and was initially treated by Resident A but was handed off after shift change to Resident B who placed a central line, both residents A and B would get credit for seeing this acutely ill patient. However, even if a patient qualified for multiple flags, they would only be matched with qualifying residents once (code available in the supplemental materials). In other words, a single patient with multiple flags might be matched to multiple residents, but only once per resident. This is similar to the attribution method we have used in previously published work.^14^,^22^ Although we sought to accurately attribute resident documentation using notes and orders, it should be noted that this approach may not capture activities not documented in the EHR, such as a senior resident providing a “fly-by consult” or other assistance to a more junior resident. We operationally defined a black-cloud resident as being in the top tertile for cohort for the entire academic year.

Because we determined our sample size by availability rather than an a priori power calculation, we conducted a post hoc power calculation that suggests we have 71% power to detect an association between being a black cloud during postgraduate year one (PGY 1) and being a black cloud during PGY 3. (See Supplemental Document for power calculation.)

### Statistical Methods

#### Weighted Kappa

For the primary analysis, the count outcome of “acutely ill patients seen” was categorized into tertiles to improve interpretability. The primary statistical analysis compares the correspondence between being in the top tertile of residents during PGY 1 with being in the top tertile during PGY 3 using a weighted kappa statistic for interrater reliability, where the “raters” in this case is the residents’ tertile rank during PGY 1 and PGY 3. This identifies whether cloud color was a stable attribute maintained during a trainee’s entire residency—as the superstition suggests. More importantly, tertile grouping provides a more actionable grouping for the pragmatic aim of this work: identifying EM residents at risk of having insufficient exposure to acutely ill patients during their residency.

#### Linear Regression

While the weighted kappa analysis is highly interpretable, using tertiles risks coarsens the metrics and potentially obfuscates the association. To complement this approach, we performed a linear regression evaluating number of acutely ill patients seen as a PGY-3 resident as a function of acutely ill patients seen in previous years:


$$Y_{3i}=\beta_{0}+\beta_{1}Y_{1i}+\beta_{2}Y_{2i}+{ɛ}_{i}$$

where *Y_ji_* is the count of acutely ill patients seen in year *j* by resident *i*.

For both the weighted kappa and linear regression analyses, data were aggregated by year, which mitigated confounding from shift distribution and rotations throughout the year.

#### Subgroup and Sensitivity Analyses

Because graduates of our residency program often pursue further training, residents interested in critical care may intentionally seek out acutely ill patients. Therefore, we performed a subgroup analysis to compare residents who entered a critical care fellowship directly after graduation with all other residents.

While the COVID-19 pandemic disrupted training environments, we minimized impact on our primary findings by evaluating relative tertile differences rather than absolute changes over time. Nevertheless, operational disruptions during 2020 and 2021 had varied manifestations; therefore, we reran the primary (weighted kappa) analysis without the cohorts who finished residency in 2020 and 2021 to confirm that results were not affected by the pandemic and associated disruptions to ED care.^23^–^25^

Finally, we augmented our linear model to adjust for overall patient volume during PGY 3 to ensure differences in acutely ill patients seen was not primarily a function of differences in overall resident throughput.

#### Missing Data Handling

It is likely that some EHR data were missing, such that our definition of acutely ill patients was not perfectly sensitive or some patient encounters may not have been attributed to the residents who provided their care. We assumed that missing data occurred uniformly due to systemic features (eg, shift timing or crowding). Because these features were equally distributed across residents, the data were pooled for aggregation.

## RESULTS

A total of 93 residents met the criteria for inclusion. Residents saw an average (standard deviation [SD]) of 167.5 (19.8) acutely ill patients during their residency. As shown in Table 2, this increased sharply by year, with PGY-1 residents seeing an average of 23.8 (7.3) acutely ill patients, PGY-2 residents an average of 43.3 (9.0), and PGY-3 residents an average of 100.3 (19.4). Trajectories of residents by month of residency are presented in Figure 1.

The counts of patients identified as acutely ill and treated by residents are in Table 1. Note that because patients could have qualified for multiple criteria (eg, ESI-1, received vasopressors, and admitted to ICU), the sum of this table does not match the resident-specific totals noted above.

Average number of acutely ill patients seen by residency year is shown in Table 2. In our primary analysis, we found a weighted κ association of −0.274 (ie, a negative agreement between intern and senior residency years for being in the top tertile for seeing acutely ill patients), shown in Table 3. These results were corroborated by our linear analysis of continuous data (Table 4), which also found a negative association between seeing more acutely ill patients as a PGY-1 resident with seeing additional patients as a PGY-3 resident, relative to the overall average. The coefficient of interest—*PGY-1 count of* acutely *ill patients—*was 0.84 (95% CI, −1.4 to −0.31), suggesting that for every acutely ill patient seen during intern year, that resident saw 0.84 fewer acutely ill patients than average their final year of residency.

Of the six residents who pursued a fellowship in critical care, only 33% were in the top tertile of residents during their PGY 1, and only 50% were in the top tertile of residents their final year (PGY 3).

### Sensitivity Analyses

Results of both the weighted kappa and linear regression analyses were fundamentally unchanged when excluding COVID-19–affected years, suggesting our results were robust to the operational disruptions posed by the pandemic. Our findings were also robust to overall patient throughput. It is possible for residents to see more acutely ill patients by seeing more patients overall. However, the negative association between acutely ill patients seen during PGY 1 and those seen in PGY 3 persists. The details of both sensitivity analyses are in the Supplemental Document.

## DISCUSSION

We do not see evidence that EHR-documented treatment of acutely ill patients—a working definition for “cloud type”—is a stable trait over time for EM residents. Among all residents, there was a negative association between being in the top (third) tertile for treating acutely ill patients as a PGY-1 resident with being the top tertile as a PGY-3 resident. This was true for residents in general as well as those who pursued a fellowship in critical care. Figure 2 shows that there is a great deal of movement between tertiles across years. Table 2 demonstrates a substantial longitudinal increase in both the absolute number and proportion of acutely ill patients seen by residents. This trend reflects changes in the resident shifts and expectations as residents progress—more adult ED shifts, more trauma shifts, and increased patient throughput expectations—which is why all analyses are relative within a residency cohort.

Our finding is consistent with previous studies that found no association between residents’ perceived cloud-type (assessed with many different instruments and operational definitions) and objective measures of workload.^9^,^12^,^26^ It is possible that there may be a tendency that once a resident thinks they have seen a lot of acutely ill patients and that perception is reinforced by peers and faculty through confirmation bias, there is less urgency to maintain that perception. Conversely, residents who view themselves as white clouds may actively seek out acutely ill patient encounters to increase their exposure to real or perceived deficits in their medical experience. Despite the level of movement overall, 2/93 residents (2.15%) stayed in the top tertile for all three PGYs, and 3/93 (3.23%) stayed in the bottom tertile for all three PGYs. Both proportions are less than would be expected by chance (albeit not statistically significantly, see supplemental document for calculations), which suggests there could be some level of intention by residents regarding the number of acutely ill patients they treat. An alternative explanation would also be regression to the mean, which would explain our observed data without requiring intentional action by residents. However, the high level of dispersion across all tertiles shown in Figure 2 suggests that regression to the mean is an incomplete explanation. More work is needed to understand the reason for these shifts in behavior.

While most residents appear to move around the tertiles of acutely ill patient exposure, it is still important to identify those residents who remain in the bottom tertile for targeted additional educational opportunities (eg, critical care simulation). Precision education and targeted learning opportunities are a pillar of competency-based medical education,^27^ and the EHR has previously been shown in emergency medicine to be useful for identifying potential training deficits and highlighting opportunities for informed self-assessment and improved goal setting.^15^

One might expect residents interested in critical care to be more inclined to seek out acutely ill patients while working in the ED. However, we did not find evidence that critical care-interested residents see a greater proportion of these patients, although the limited number of residents pursuing a critical care fellowship prevents further generalization. We surmise that this finding could also indicate that critical care-bound residents find a way to be involved with these cases in a teaching role instead, mentoring junior residents through the cases, which would not be reflected in our data due to our method of attributing patients via the EHR. This is an area for future study, ideally with a larger pool of critical care-bound residents from multiple academic centers.

## LIMITATIONS

This study has several limitations. First, it was conducted at a single academic medical center with a three-year residency. While residency length alone is unlikely to substantially influence these findings, the culture and catchment area of this center may not be representative of other residency programs and patient populations.

Second, some acutely ill EHR phenotypes may not be obvious at the time of a resident’s decision to sign up for a patient. Some phenotypes, especially patients triaged as ESI 1, are clearly indicated in the EHR at the time the resident signs up for the patient. Others, like those identified as acutely ill by diagnosis, would not have met our study criteria at the time the resident assumed care. This would be a limitation for any causal analysis where the unit of analysis was the patient; however, in the real world setting of the ED, we believe it is a reasonable assumption that residents generally recognize whether they are assuming care of an acutely ill patient. For example, a resident who signs up for a patient who will eventually be diagnosed for anaphylaxis compared to a sprained wrist is likely to recognize whether they are signing up for an acutely ill patient.

Third, this analysis does not address the quality of care. It could be that the residents seeing the fewest acutely ill patients are nevertheless doing the best job at caring for those patients. This is very likely the case for some of the lower volume residents who pursue a critical care fellowship. Additionally, it is not clear what level of clinical experience defines competency; it is possible that all residents far exceed (or undershoot) the bar for minimum competence in caring for critical patients. More research on competency and clinical exposure with a more comprehensive dataset including patient outcomes is indicated.

Fourth, we do not compare our objective measure (tertiles of acutely ill patients cared for) to perceived measures of black versus white clouds, either by residents’ self-assessments or by residents rating each other’s “cloud type.” This also remains an area for subsequent study. Fifth, in the absence of a clear, gold standard definition of “acutely ill,” we made our own definition based on available data and attending physician experience. It is possible that patients who were acutely ill were not captured by our definition, but we believe this definition has construct validity and matches the experience of residents at the study institution, although it should be acknowledged that this definition is contingent on the documentation practices of the study institution and may not have strong external validity at other institutions.

Finally, we used EHR data for this study. Electronic health record data are objective and clearly important in medical care today. However, they do not necessarily reflect the whole experience of residents. Expanding this work by collaborating with other institutions to increase the sample size and variability of resident experiences would help to address many of these limitations and remains a hoped-for future direction. Increasing the pool of residents and data would also allow for analysis of tail-end effects, such as whether the “darkest” or “whitest” clouds (eg, 1^st^ or 10^th^ decile) are more or less likely to change cloud group than their peers in the “messy middle.”

## CONCLUSION

We did not find evidence that high or low EHR-attributed exposure to acutely ill adult ED patients is a stable, resident-specific trait over the course of EM residency. This finding questions the narrative that some resident physicians are “black clouds” or “white clouds” who seem to attract or deflect the most serious cases in the ED. Residents who start off seeing many acutely ill patients often end up seeing fewer of those patients than their peers later in residency. Residency programs may benefit from considering the percentage of acutely ill patients that their residents see as a part of providing precision education.

## Supplementary Information



## Figures and Tables

**Figure 1:**
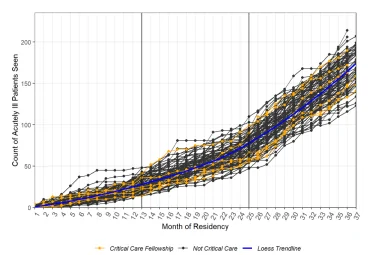
Cumulative trajectories of acutely ill patients seen by resident during the course of a three-year emergency medicine residency.

**Figure 2:**
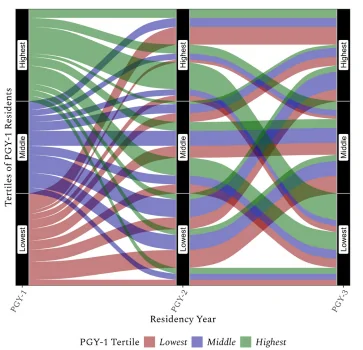
Alluvial plot of resident tertile rankings by postgraduate year for a study of acutely ill patients seen during residency. *PGY*, postgraduate year.

**Table 1:** Distribution of acutely ill patients seen by residents by indication in a study of the concordance of acutely ill patients seen by residents by postgraduate year.

Acute Patient	Total Patients	Average Patients per Resident
Anaphylactic shock (diagnosis)	484	5.20
Arterial line placement (procedure order)	244	2.62
Central line placement (procedure order)	211	2.27
Death in ED (patient record)	308	3.31
ESI 1-Red (triage)	4,681	50.33
Admission to ICU (ADT Documentation)	9,656	103.83
IV insulin drip given (medication)	534	5.74
IV ketamine drip given (medication)	17	0.18
Level I trauma (flowsheets)	4,014	43.16
STEMI (diagnosis)	1,589	17.09
Vasodilators given (medication)	331	3.56
Vasopressors given (medication)	750	8.06

*ADT*, admission, discharge, transfer; *ED*, emergency department; *ESI*, Emergency Severity Index; *ICU*, intensive care unit; *IV*, intravenous; *STEMI*, ST-elevation myocardial infarction.

**Table 2:** Center and spread of acutely ill patients seen by residency year.

Residency Year	Count of Acutely Ill Patients Seen						Acutely Ill Proportion of All Patients Seen		
	Minimum	Maximum	Mean	Standard Deviation	Median	Interquartile Range	Minimum	Maximum	Mean
PGY-1	11	47	23.8	7.3	23	(18.0–29.0)	1.47%	4.48%	2.86%
PGY-2	25	62	43.3	9	44	(37.0–49.0)	2.56%	5.78%	3.78%
PGY-3	59	150	100.3	19.4	101	(86.0–114.0)	4.18%	10.85%	6.64%

*PGY*, postgraduate year.

**Table 3:** Concordance of being in the top tertile for acutely ill patients seen across residency by postgraduate year.

	PGY-3 Tertile			Total
	Tertile 1 (Lowest)	Tertile 2	Tertile 3 (Highest)	
PGY-1 tertile				
Tertile 1 (lowest)	7	11	13	31
Tertile 2	7	12	12	31
Tertile 3 (highest)	17	8	6	31
Total	31	31	31	93

*PGY*, postgraduate year

**Table 4:** Linear regression results of acutely ill patients seen in final year of residency for a study of the concordance of acutely ill patients seen by residents by postgraduate year.

Characteristic	Beta	95% CI	*P* value
(Intercept)	128	107, 149	< .001
PGY-1 count of acutely ill patients	−0.84	−1.4, −0.31	.002
PGY-2 count of acutely ill patients	−0.18	−0.61, 0.26	.40

*PGY*, postgraduate year.
