# Supplementary material for: Resident Exposure to Acutely Ill Patients Over the Course of Residency
Source: West J Emerg Med. 2026 Sep 24;27(5):1235–42. doi: 10.5811/westjem.63704 (PMC13660598; doi:10.5811/westjem.63704)
Supplement: Supplementary file 1 [file wjem-27-1235-s001.docx]

What Color is Your Cloud? Trajectory of Cummulative Acutely Ill Patients Seen Over the Course of Residency

A retrospective cohort analysis

Daniel J Hekman

Joe-Ann S Moser

Benjamin H Schnapp

Table of contents

[Question Formulation	2](#_Toc234003799)

[Pre-Introduction	3](#_Toc234003800)

[STROBE 1: Title and Abstract	3](#_Toc234003801)

[Abstract summary	3](#_Toc234003802)

[Introduction	3](#_Toc234003803)

[STROBE 2: Background and rationale	3](#_Toc234003804)

[STROBE 3: State objectives	3](#_Toc234003805)

[Methods	4](#_Toc234003806)

[STROBE 4: Study Design	4](#_Toc234003807)

[STROBE 5: Setting	4](#_Toc234003808)

[STROBE 6: Participants	4](#_Toc234003809)

[STROBE 7: Variables	5](#_Toc234003810)

[Primary Outcome	5](#_Toc234003811)

[Resident Attribution	5](#_Toc234003812)

[STROBE 8: Data Sources	6](#_Toc234003813)

[STROBE 9: Bias	6](#_Toc234003814)

[STROBE 10: Study size determination	6](#_Toc234003815)

[Power Calculation (even if post-hoc) with pwrss package.	6](#_Toc234003816)

[STROBE 11: Quantitative variables handling	7](#_Toc234003817)

[STROBE 12: Statistical methods	7](#_Toc234003818)

[STROBE 12a: Methods	8](#_Toc234003819)

[STROBE 12b: Subgroup and interaction methods	8](#_Toc234003820)

[STROBE 12c: Missing data handling	9](#_Toc234003821)

[STROBE 12d: Design-specific items	9](#_Toc234003822)

[STROBE 12e: Sensitivity analyses	9](#_Toc234003823)

[Load Code	9](#_Toc234003824)

[Results	11](#_Toc234003825)

[STROBE 16: Main Results	16](#_Toc234003826)

[Categorical Stats by Tertile	16](#_Toc234003827)

[Continuous Stats	18](#_Toc234003828)

[Linear Regression	24](#_Toc234003829)

[STROBE 17: Other analyses	25](#_Toc234003830)

[Top or Bottom All the way through	27](#_Toc234003831)

[Sensitivity analysis: COVID	28](#_Toc234003832)

[Sensitivity: Adjusting for total patients seen.	30](#_Toc234003833)

[Discussion	32](#_Toc234003834)

[STROBE 18: Key results summary	32](#_Toc234003835)

[STROBE 19: Limitations	33](#_Toc234003836)

[STROBE 20: Interpretation	33](#_Toc234003837)

[STROBE 21: Generalizability	33](#_Toc234003838)

[STROBE 22 Funding source and role	33](#_Toc234003839)

[Save images	33](#_Toc234003840)

# Question Formulation

What is the PICOT Question?

- **Popuation**: Emergency Medicine Resident Physicians
- **Intervention/Exposure**: Time - month of residency
- **Comparison**: Other RMDs in the same program
- **Outcome**: Number of Acutely Ill Patients seen
- **Timeframe**: Over the Course of the porgram: measures at end of intern year, end of program

What is the correlation between being a “Black Cloud Resident”, defined as top tertile of acutely ill patients seen, at the end of intern year and at the end of the program? Do some people just see more acutely ill patients throughout residency, or is it more random?

# Pre-Introduction

## STROBE 1: Title and Abstract

What Color is Your Cloud? Trajectory of Cumulative Acutely Ill Patients Seen Over the Course of Residency: A retrospective cohort analysis

### Abstract summary

**Introduction**

**Methods**

**Results**

**Discussion**

# Introduction

## STROBE 2: Background and rationale

Emergency Medicine (EM) physicians have long been superstitious about the mysterious ebbs and flows of their practice environment. Empirical studies of these phenomena have failed to find significant, measurable differences in clinical volumes or patient acuity to back up these superstitions.

Another superstition that is pervasive across medicine may have a more solid empirical footing is the idea that individuals may represent “black clouds” or “white clouds”, seeing a higher or lower proportion of patients or of critical illness compared to other physicians.

The “black cloud”/“white cloud” phenomenon may have important implications for clinical training; by the time of graduation, EM residents were found to have significant differences in their experience. Experiential learning theory suggests that clinical exposures are essential for growth by allowing residents to actively experiment with learned knowledge and critically reflect on what happened. “White cloud” residents with less clinical exposure to acutely ill patients than their cohort may benefit from additional opportunities to simulate these cases or even from additional rotations in the ICU to bolster their experience with the management of critically ill patients.

However, to our knowledge, no study has looked at variability in EM resident clinical exposure to acutely ill patients during their EM training.

## STROBE 3: State objectives

The goal of this study was to describe the distribution of acutely ill patients seen by residents over the course of their residency. We then use this distribution to determine whether some residents saw a significantly higher or lower number of acutely ill patients than other trainees and whether that difference was stable over time–i.e., if some residents are “black clouds” who consistently see the sickest patients. We also look at whether residents who go on to do a critical care fellowship are more likely to have consistently treated acutely ill patients than their peers.

Objective 1: Quantify the distribution (at a single institution) of variability in How many critically ill patients residents see.

Objective 2: Track if “Gets lots of crazy cases” is a stable attribute over time (PGY1 vs PGY3) for residents.

Objective 3: Identify if CC-followship-bound RMDs are more likely to have consistently taken on acutely ill patients (i.e., to be “black clouds”).

# Methods

## STROBE 4: Study Design

A retrospective cohort analysis

## STROBE 5: Setting

This retrospective cohort study uses data from EM residents at an ED that treats approximately 70,000 patients per year and is associated with an academic medical system and level I trauma center in the Midwestern United States. The ED has both pediatric and general pods with separate faculty, and residents work shifts in both.

## STROBE 6: Participants

This program hosts a three-year residency program that, since 2023, accepts 13 residents per year (2015 through 2022, classes were 12 residents). We extracted 11 years of electronic health record (EHR) data, July 2015 through June 2025, representing 9 complete cohorts of EM Residents.

This was a retrospective review of data that was stored on secure storage, and this study was approved as a quality improvement initiative by our institution’s health sciences institutional review board because it was part of a larger effort on improving data-driven feedback for residents. All data retrieval and processing was done using SQL and R.@rcoreteamLanguageEnvironmentStatistical2024

## STROBE 7: Variables

### Primary Outcome

Our primary outcome of interest was the number of acutely ill patients seen by each resident during each year of their residency. A patient was identified as acutely ill if they met any of the following criteria (documentation source):

- ESI 1-Red (triage)
- Admission to ICU (ADT Documentation)
- Level I Trauma (triage)
- STEMI (diagnosis)
- Death in ED (patient record)
- Central Line Placement (procedure order)
- Arterial Line Placement (procedure order)
- Vasopressors given (medication)
- Vasodilators given (medication)
- Anaphylaxis Shock (diagnosis)
- Ketamine given (medication)
- IV Insulin given (medication)

### Resident Attribution

Resident providers are matched with acutely ill patient care in three ways, depending on the type of acute illness flag. For medication-based flags, attribution was based on the resident who was the ordering provider for the medication. For procedure-based flags, attribution was based on the resident was the performing provider listed on the procedure order in the EHR. For all other flags, resident attribution was based on the provider to first sign the primary ED Provider Note. Thus, it was possible for the same patient to be attributed to multiple residents if the patient met multiple flags with different attributions–for example, a patient who arrived as a level 1 trauma and was initially treated by Resident A, but was handed off after shift change to Resident B who placed a central line, both residents A and B would get credit for seeing this acutely ill patient.

We include all residents for whom we have 36 months of data in the EHR. This excludes patients who took time off during residency and graduated off cycle; current residents; and residents who were present during the EHR implementation (2014). Although sample size was chosen based on availability rather than an *ex-ante* power calculation of the number of residents and patients needed, a post-hoc power calculation suggests that we have 71% power to detect an association between being a “Black cloud” during intern year (operationally defined as being in the top tertile for cohort) and being a black cloud as a PGY-3 resident (See Supplemental Document for power calculation).

## STROBE 8: Data Sources

EHR Data

## STROBE 9: Bias

## STROBE 10: Study size determination

**How did we arrive at Sample Size?**

Including all residents for whom we have 36 months of data in the EHR. This excludes patients who took time off during residency and graduated off cycle; current, ungraduated residents; and residents who were present during the EHR implementation (2014), for whom data are incomplete.

### Power Calculation (even if post-hoc) with pwrss package.

Using a floor estimate of $\chi^{2}$ test statistic from results.

```
pwrss::power.chisq.test(
    ncp = 10,
    df = 4, #3x3 table
    alpha = 0.05,
    plot = TRUE
)
```

```
+--------------------------------------------------+
|                POWER CALCULATION                 |
+--------------------------------------------------+

Generic Chi-square Test

---------------------------------------------------
Hypotheses
---------------------------------------------------
  H0 (Null Claim)   : ncp = null.ncp
  H1 (Alt. Claim)   : ncp > null.ncp

---------------------------------------------------
Results
---------------------------------------------------
  Type 1 Error (alpha)   = 0.050
  Type 2 Error (beta)    = 0.284
  Statistical Power      = 0.716  <<
```

Although sample size was chosen based on availability rather than an ex-ante power calculation of the number of residents and patients needed, a post-hoc power calculation suggests that we have 71% power to detect an association between being a “Black cloud” during PGY-1 (operationally defined as being in the top tertile for cohort) and being a black cloud as a PGY-3 resident.

## STROBE 11: Quantitative variables handling

For the primary analysis, the countable outcome of “Acutely Ill Patients Seen” is bucketed into tertiles, which is consisent with our department education team’s review of residents.

## STROBE 12: Statistical methods

Weighted Kappa

For the primary analysis, the countable outcome of “Acutely Ill Patients Seen” is bucketed into tertiles, which is consistent with our department education team’s review of residents. The primary statistical analysis compares the correspondence between being in the top tertile of residents during the intern year with being in the top tertile during PGY-3 using a weighted $k$ statistic for interrater reliability. This will identify if cloud color is a “sticky” attribute that is maintained during one’s entire residency–as the superstition suggests.

The primary statistical analysis compares the correspondence between being in the top tertile of residents during the intern year with being in the top tertile during PGY-3 using a weighted $\kappa$ statistic for interrater reliablility. This will identify if cloud color is a “sticky” attribute that is maintained during one’s entire residency–as the superstition suggestions–or not.

### STROBE 12a: Methods

Describe all statistical methods, including those used to control for confounding

Over the course of each year, we expect all major confounding variables such as shift distribution, patient type by seasonality (e.g. “respiratory season” in the winter and “trauma season” in the summer) distribution, day vs night shifts, etc. to be evenly distributed across all residents in a given cohort based on the scheduling process for residents.

While the weighted $\kappa$ analysis aligns with our internal processes for resident review and is very interpretable, the use of tertiles risks coarsening our metrics and potentially obfuscating some elements of the association. So to expand our primary analysis through a different statistical lens, we also performed a linear regression for number of acutely ill patients seen as a senior resident as a function of acutely ill patients seen as an intern. We also generate two data visualizations to visually elucidate the trend of acutely ill patients seen over the course of residency. We use a linear model to adjust for overall patient volume to make sure we are not just capturing differences overall resident throughput.

For both the weighted $\kappa$ and linear regression analyses, data are aggregated by year, which will mitigate confounding shift distribution and rotations throughout the year. Unfortunately, it may not completely control for any order effects of having specific rotations (e.g. trauma, anesthesia) early or late during intern year. These effects, if any, are being evaluated by **Kim et al, in press.**.

The COVID-19 pandemic, while disruptive, should not have affected our primary results because we are comparing relative differences by tertile rather than absolute changes over time. Nevertheless, operational disruptions during 2020 and 2021 had varied manifestations, so we reran the primary (weighted $\kappa$) analysis without the cohorts who finished residency in 2020 and 2021 to confirm that results were not affected by the COVID-19 Pandemic and associated disruptions to ED care.@waiChangesEmergencyDepartment2022;**^phendEmergencyDepartmentVolume2023?^**;**^heppnerCOVID19EmergencyDepartment2021?^**

### STROBE 12b: Subgroup and interaction methods

Critical Care Fellowship-bound residents vs. not.

Because our residency program is three years, it is common for graduates to do a fellowship, and it seems plausible that residents who choose to do a fellowship in critical care are different from their peers in their approach to seeking out acutely ill patients to treat, so we do a subgroup analysis comparing future critical care fellows to all other residents.

### STROBE 12c: Missing data handling

It is likely that some EHR data was missing such that our definition of acutely ill patients was not perfectly sensitive or our attributions of patients to a resident may have missed some patients cared for by our residents. However, we assume, based on experience with EHRs, that these missing data are missing at random, or at least missing based on systemic features that are equally distributed across all residents in a given year and thus are safely aggregated over. Residents with missing data for large swaths of an entire year due to extended leave and off-cycle graduation were excluded.

### STROBE 12d: Design-specific items

N/A

### STROBE 12e: Sensitivity analyses

COVID - Remove COVID years.

Adjusting for total patients seen - Is “Number of black cloud patients seen” just a proxy for “resident PPH/Throughput”?

# Load Code

```
options(tidyverse.quiet = TRUE)
library(tidyverse)
library(RSQLite)
edu_db <- dbConnect(SQLite(), uwdem.pkg::uwdem_random_rds_dir('Education', 'Resident Dashboards', 'Data', 'Resident_DB_Data.sqlite'))
bc_df <- dbReadTable(edu_db, 'black_cloud_pats') |>
        mutate(
            across(ends_with('dttm'), as.POSIXct),
            across(ends_with('time'), as.POSIXct),
            across(ends_with('inst'), as.POSIXct),
            across(ends_with('date'), as.Date),
        )
dbDisconnect(edu_db)

# Load fellows by internal ID rather than storing names here
future_cc_fellow_ids <- c(
    235149,
    235174,
    234920,
    235047,
    234528,
    234579
)
future_cc_fellows <- bc_df |>
    select(treating_resident_id, treating_resident) |>
    distinct() |>
    filter(treating_resident_id %in% future_cc_fellow_ids) |>
    pull(treating_resident)

theme_bc_paper <- function() {
    gg_functions <- list(
        ggplot2::theme_bw(),
        ggplot2::theme(
            text = element_text(
                family = 'Proxima Nova',
                color = 'black'
            ),
            legend.text = element_text(family = 'Proxima Nova', face = 'italic'),
            plot.title = element_text(family = 'Spectral', face = 'bold'),
            axis.title =  element_text(family = 'Spectral'),
            axis.text.x = ggplot2::element_text(angle = 60, size = 10, hjust = 1),
            panel.grid.minor = ggplot2::element_blank(),
            legend.position = 'bottom',
            plot.title.position = 'plot',
            plot.caption.position = 'plot'
        )
    )
}
bc_by_resident_by_month <- bc_df |>
    mutate(
        # correction for June start
        month = floor_date(treating_resident_dttm, 'month'),
        first_month_of_res = min(month),
        month = case_when(
            month(month) == 6 &
            month == first_month_of_res ~ month + months(1),
            .default = month
        )
    ) |>
    pivot_longer(
        cols = starts_with('bc_'),
        names_to = "blackcloud_qualifier"
    ) |>
    mutate(value = coalesce(value, 0)) |>
    group_by(
        resident = treating_resident,
        month,
        pat_enc_csn_id
    ) |>
    summarise(
        blackcloud_patient = max(value, na.rm = TRUE),
        .groups = 'drop_last'
    ) |>
    summarise(
        all_patients = n(),
        blackcloud_patients = sum(blackcloud_patient),
        .groups = 'drop'
    ) |>
    group_by(resident) |>
    tidyr::complete(
        month = seq.Date(
            from = as.Date(min(month)),
            to = as.Date(max(month)),
            by = 'month'),
        fill = list(
            blackcloud_patients = 0,
            all_patients = 0
        )
    ) |>
    arrange(month) |>
    mutate(
        r = row_number(),
        n_months = max(r),
        cumulative_bcs = cumsum(blackcloud_patients),
        cumulative_pats = cumsum(all_patients)
    ) |>
    mutate(
        future_critcare_fellow = resident %in% future_cc_fellows
    ) |>
    filter(
        n_months %in% c(35:37) &
        sum(all_patients) > 1000
    )
```

# Results

```
trajectories_df <- bc_by_resident_by_month |>
    mutate(
        future_critcare_fellow = as.numeric(future_critcare_fellow)
    ) |>
    arrange(
        future_critcare_fellow,
        resident,
        month
    )

gg_fig_trajectories <- trajectories_df |>
    ggplot(aes(
        x = r,
        y = cumulative_bcs
    )) +
    geom_point(
        data = dplyr::filter(trajectories_df, future_critcare_fellow == 0),
        aes(color = factor(
            future_critcare_fellow,
            levels = c(1, 0),
            labels = c('Critical Care Fellowship', 'Not Critical Care')
        ))) +
    geom_line(
        data = dplyr::filter(trajectories_df, future_critcare_fellow == 0),
        aes(group = resident,
        color = factor(
            future_critcare_fellow,
            levels = c(1, 0),
            labels = c('Critical Care Fellowship', 'Not Critical Care')
        )
    )) +
    geom_point(
        data = filter(trajectories_df, future_critcare_fellow == 1),
        aes(color = factor(
            future_critcare_fellow,
            levels = c(1, 0),
            labels = c('Critical Care Fellowship', 'Not Critical Care')
        ))) +
    geom_line(
        data = filter(trajectories_df, future_critcare_fellow == 1),
        aes(group = resident,
        color = factor(
            future_critcare_fellow,
            levels = c(1, 0),
            labels = c('Critical Care Fellowship', 'Not Critical Care')
        )
    )) +
    stat_smooth(
        aes(color = 'Loess Trendline'),
        formula = y ~ x,
        method = loess,
        se = FALSE
    ) +
    geom_vline(
     data = tibble(x = c(13,25,37)),
     aes(xintercept = x)
    ) +
    scale_x_continuous(
        name = 'Month of Residency',
        breaks = c(1:37),
        limits = c(1, 37),
        expand = expansion(0)
    ) +
    scale_y_continuous(
        name = 'Count of Acutely Ill Patients Seen',
        expand = expansion(mult = c(0, 0.1))
    ) +
    scale_color_manual(
        name = '',
        values = c(
            'Critical Care Fellowship' = 'orange',
            'Not Critical Care' = 'gray20',
            'Loess Trendline' = 'blue'
        ),
        breaks = c(
            'Critical Care Fellowship',
            'Not Critical Care',
            'Loess Trendline'
        )
    ) +
    theme_bc_paper()
gg_fig_trajectories
```

Figure 1: Trajectories of Cumulative Critically Ill Patients Seen by Resident

```
by_resident_year <- bc_by_resident_by_month |>
    group_by(
        pgy = case_match(r,
            c(1:12) ~ 'Intern',
            c(13:24) ~ 'PGY2',
            c(25:37) ~ 'Senior'
        ),
        resident
    ) |>
    summarise(
        all_pts_this_year = sum(all_patients),
        all_bc_pts_this_year = sum(blackcloud_patients),
        cummulative_bc_pats = max(cumulative_bcs),
        .groups = 'drop_last'
    )
by_year <- by_resident_year |>
    summarise(
        min_bc_pats = min(all_bc_pts_this_year),
        max_bc_pats = max(all_bc_pts_this_year),
        mean_bc_pats = mean(all_bc_pts_this_year),
        sd_bc_pats = sd(all_bc_pts_this_year),
        median_bc_pats = median(all_bc_pts_this_year),
        q1_bc_pats = quantile(all_bc_pts_this_year, p = 0.25),
        q3_bc_pats = quantile(all_bc_pts_this_year, p = 0.75),
        p_min_bc_pats = min(all_bc_pts_this_year / all_pts_this_year),
        p_mean_bc_pats = mean(all_bc_pts_this_year / all_pts_this_year),
        p_max_bc_pats = max(all_bc_pts_this_year / all_pts_this_year),
        .groups = 'drop'
    )

bc_overall_meansd <- bc_by_resident_by_month |>
    ungroup() |>
    filter(r == n_months) |>
    summarise(
        mean_bc_pats = mean(cumulative_bcs),
        sd_bc_pats = sd(cumulative_bcs)
    ) |>
    mutate(across(ends_with('bc_pats'), \(x) scales::number(x, accuracy = 0.1)))

res <- list(
    n_residents = n_distinct(bc_by_resident_by_month$resident),
    by_year = by_year |>
        mutate(across(ends_with('bc_pats'), \(x) scales::number(x, accuracy = 0.1))) |>
        nest(meansd = c(mean_bc_pats, sd_bc_pats)) |>
        tidyr::drop_na() |>
        tibble::column_to_rownames('pgy')
) |> append(
    bc_overall_meansd |> as.list()
)
```

Table 1

tbl_qual_tbl <- bc_df |>
 filter(treating_resident %in% bc_by_resident_by_month$resident) |>
 pivot_longer(
 cols = starts_with('bc_'),
 names_to = "blackcloud_qualifier"
 ) |>
 mutate(value = coalesce(value, 0)) |>
 group_by(
 blackcloud_qualifier,
 treating_resident_id
 ) |>
 summarise(
 patients_seen = sum(value, na.rm = TRUE),
 .groups = 'drop_last'
 ) |>
 summarise(
 total_patients_seen = sum(patients_seen),
 avg_patients_seen = mean(patients_seen)
 ) |>
 mutate(`Acute Patient` = case_match(blackcloud_qualifier,
 'bc_anaphylaxis' ~ 'Anaphalaxis Shock (diagnosis)',
 'bc_arterial_line' ~ 'Arterial Line Placement (procedure order)',
 'bc_central_line' ~ 'Central Line Placement (procedure order)',
 'bc_death_in_ed' ~ 'Death in ED (patient record)',
 'bc_esi1' ~ 'ESI 1-Red (triage)',
 'bc_icu' ~ 'Admission to ICU (ADT Documentation)',
 'bc_iv_insulin' ~ 'IV Insulin drip given (medication)',
 'bc_ketamine' ~ 'IV Ketamine drip given (medication)',
 'bc_lvl1trauma' ~ 'Level I Trauma (flowsheets)',
 'bc_stemi' ~ 'STEMI (diagnosis)',
 'bc_vasodilators' ~ 'Vasodilators given (medication)',
 'bc_vasopressors' ~ 'Vasopressors given (medication)'
 ), .before = 1
 ) |>
 filter(!is.na(`Acute Patient`)) |>
 select(-blackcloud_qualifier) |>
 gt::gt() |>
 gt::cols_label(.list = list(
 total_patients_seen = 'Total Patients',
 avg_patients_seen = 'Average Patients per Resident'
 )) |>
 gt::fmt_number(
 columns = total_patients_seen,
 decimals = 0,
 use_seps = TRUE
 ) |>
 gt::fmt_number(
 columns = avg_patients_seen,
 decimals = 2,
 use_seps = TRUE
 )

## STROBE 16: Main Results

### Categorical Stats by Tertile

```
by_resident  <- bc_by_resident_by_month |>
    group_by(
        pgy = case_match(r,
            c(1:12) ~ 'Intern',
            c(13:24) ~ 'PGY2',
            c(25:37) ~ 'Senior'
        ),
        resident,
        future_critcare_fellow
    ) |>
    summarise(
        all_pts_this_year = sum(all_patients),
        all_bc_pts_this_year = sum(blackcloud_patients),
        cummulative_bc_pats = max(cumulative_bcs),
        .groups = 'drop'
    )

with_tertiles <- by_resident |>
    group_by(pgy) |>
    mutate(
        n_residents = n(),
        rank = row_number(all_bc_pts_this_year),
        tertile = ntile(all_bc_pts_this_year, 3)
    ) |>
    pivot_wider(
        id_cols = resident,
        names_from = pgy,
        names_prefix = 'tertile',
        names_sep = '_',
        values_from = tertile
    ) |>
    janitor::clean_names() |>
    labelled::set_variable_labels(
        tertile_intern = 'PGY-1 Tertile',
        tertile_senior = 'PGY-3 Tertile'
    )


table(with_tertiles$tertile_intern, with_tertiles$tertile_senior) |>
    chisq.test()

    Pearson's Chi-squared test

data:  table(with_tertiles$tertile_intern, with_tertiles$tertile_senior)
X-squared = 10.065, df = 4, p-value = 0.03935
primary_result <- table(with_tertiles$tertile_intern, with_tertiles$tertile_senior) |>
    psych::wkappa()

table(with_tertiles$tertile_intern, with_tertiles$tertile_senior) |>
    psych::cohen.kappa(n.obs = nrow(with_tertiles))
Call: cohen.kappa1(x = x, w = w, n.obs = n.obs, alpha = alpha, levels = levels,
    w.exp = w.exp)

Cohen Kappa and Weighted Kappa correlation coefficients and confidence boundaries
                 lower estimate  upper
unweighted kappa -0.23   -0.097  0.038
weighted kappa   -0.47   -0.274 -0.080

 Number of subjects = 93
res2 <- with(with_tertiles |> filter(resident %in% future_cc_fellows), {list(
    p_ccf_top_intern = scales::percent(sum(tertile_intern == 1) / length(future_cc_fellows)),
    p_ccf_top_senior = scales::percent(sum(tertile_senior == 1) / length(future_cc_fellows))
)})

with_tertiles |>
    gtsummary::tbl_cross(
        row = tertile_intern,
        col = tertile_senior
    )
```

Table 2: Concordance of being in the top tertile across residency

|  | PGY-3 Tertile | | |  |
| --- | --- | --- | --- | --- |
|  | 1 | 2 | 3 | Total |
| PGY-1 Tertile |  |  |  |  |
| 1 | 7 | 11 | 13 | 31 |
| 2 | 7 | 12 | 12 | 31 |
| 3 | 17 | 8 | 6 | 31 |
| Total | 31 | 31 | 31 | 93 |

### Continuous Stats

```
stats_descr_continuous <- by_year |>
    dplyr::mutate(
        iqr = glue::glue("({scales::number(q1_bc_pats, accuracy=0.1)}-{scales::number(q3_bc_pats, accuracy=0.1)})")
    ) |>
    dplyr::select(
        -q1_bc_pats,
        -q3_bc_pats
    ) |>
    gt::gt() |>
    gt::cols_label(.list = list(
        pgy = 'Residency Year',
        min_bc_pats = 'Minimum',
        max_bc_pats = 'Maximum',
        mean_bc_pats = 'Mean',
        sd_bc_pats = 'Standard Deviation',
        median_bc_pats = 'Median',
        iqr = 'Interquartile Range',
        p_min_bc_pats = 'Minimum',
        p_max_bc_pats = 'Maximum',
        p_mean_bc_pats = 'Mean'
    )) |>
    gt::fmt_number(
        columns = c('mean_bc_pats', 'sd_bc_pats', 'median_bc_pats'),
        decimals = 1
    )  |>
    gt::fmt_percent(
        columns = c('p_min_bc_pats', 'p_max_bc_pats', 'p_mean_bc_pats'),
        decimals = 2
    ) |>
    gt::tab_spanner(
        label = 'Count of Acutely Ill Patients Seen',
        columns = c(
            min_bc_pats,
            max_bc_pats,
            mean_bc_pats,
            sd_bc_pats,
            median_bc_pats,
            iqr
        )
    ) |>
    gt::tab_spanner(
        label = 'Acutely Ill Proportion of All Patients Seen',
        columns = c(
            p_min_bc_pats,
            p_max_bc_pats,
            p_mean_bc_pats
        )
    )
aov(all_bc_pts_this_year ~ pgy, data = by_resident_year) |> summary()
             Df Sum Sq Mean Sq F value Pr(>F)
pgy           2 293475  146737   861.6 <2e-16 ***
Residuals   276  47004     170
---
Signif. codes:  0 '***' 0.001 '**' 0.01 '*' 0.05 '.' 0.1 ' ' 1
```

 Important

This is all for the results section of the paper because having consistent and accurate numbers throughout the paper is best done by code instead of manually updating all the numbers in a manuscript. This covers

- STROBE 13: Participants
- STROBE 14: Descriptive Data
- STROBE 15: Outcome Data
- STROBE 16: Main Results

A total of 93 residents met criteria for inclusion. Residents saw an average (standard deviation [sd]) of 167.5 (19.8) acutely ill patients during their residency. This increased sharply by year, with PGY-1 residents seeing an average (sd) of 23.8 (7.3) acutely ill patients, PGY-2 residents seeing an average (sd) of 43.3 (9.0), and PGY-3 residents seeing an average (sd) of 100.3 (19.4). Trajectories of residents by month of residency are presented in Figure 1.

The counts of patients identified as critically ill and treated by residents are in Table 3. Note that because patients could have qualified for multiple criteria (e.g., ESI-1, received vasopressors, and admitted to ICU), the sum of this table do not match the resident-specific totals noted above.

Average acutely ill patients seen by residency year is shown in Table 4. In our primary analysis, we found a weighted $\kappa$ association of -0.274, i.e., a negative agreement between intern and senior residency years for being in the top tertile for seeing acutely ill patients, shown in Table 2. These results were corroborated by our linear analysis of continuous data (Table 5), which also found a negative association between seeing more critically ill patients as an PGY-1 with seeing additional patients as a PGY-3 resident, relative to the overall average. This effect was robust to the impacts of COVID-19 and to overall volumes of patients treated during PGY3, shown in the Supplemental Document.

Of the 6 residents who went on to do a fellowship in critical care, only 33% were in the top tertile of residents during their PGY-1, and only 50% were in the top tertile of residents their final year (PGY-3).

tbl_qual_tbl

Table 3: Critically Ill Indications

| Acute Patient | Total Patients | Average Patients per Resident |
| --- | --- | --- |
| Anaphalaxis Shock (diagnosis) | 484 | 5.20 |
| Arterial Line Placement (procedure order) | 244 | 2.62 |
| Central Line Placement (procedure order) | 211 | 2.27 |
| Death in ED (patient record) | 308 | 3.31 |
| ESI 1-Red (triage) | 4,681 | 50.33 |
| Admission to ICU (ADT Documentation) | 9,656 | 103.83 |
| IV Insulin drip given (medication) | 534 | 5.74 |
| IV Ketamine drip given (medication) | 17 | 0.18 |
| Level I Trauma (flowsheets) | 4,014 | 43.16 |
| STEMI (diagnosis) | 1,589 | 17.09 |
| Vasodilators given (medication) | 331 | 3.56 |
| Vasopressors given (medication) | 750 | 8.06 |

stats_descr_continuous

Table 4: Center and Spread of Acutely Ill Patients seen by Residency Year

|  | Count of Acutely Ill Patients Seen | | | | | | Acutely Ill Proportion of All Patients Seen | | |
| --- | --- | --- | --- | --- | --- | --- | --- | --- | --- |
| Residency Year | Minimum | Maximum | Mean | Standard Deviation | Median | Interquartile Range | Minimum | Maximum | Mean |
| Intern | 11 | 47 | 23.8 | 7.3 | 23.0 | (18.0-29.0) | 1.47% | 4.48% | 2.86% |
| PGY2 | 25 | 62 | 43.3 | 9.0 | 44.0 | (37.0-49.0) | 2.56% | 5.78% | 3.78% |
| Senior | 59 | 150 | 100.3 | 19.4 | 101.0 | (86.0-114.0) | 4.18% | 10.85% | 6.64% |

### Linear Regression

Question to answer: does everyone “revert to the mean” or does seeing lots of acutely ill patients previously imply that you will see more acutely ill patients in the future?

$$Y_{3i}=\beta_{0}+\beta_{1}Y_{1i}+\beta_{2}Y_{2i}+\epsilon_{i}$$

Where $Y_{ji}$ is the count of acutely ill patients seen in year $j$ by resident $i$.

```
lin_model <- lm(
    formula = 'Senior ~ Intern + PGY2',
    data = by_resident |>
        tidyr::pivot_wider(
            id_cols = resident,
            names_from = pgy,
            values_from = all_bc_pts_this_year
        )
)

gtsummary::tbl_regression(lin_model, intercept = TRUE)
```

Table 5

| **Characteristic** | **Beta** | **95% CI** | **p-value** |
| --- | --- | --- | --- |
| (Intercept) | 128 | 107, 149 | <0.001 |
| Intern | -0.84 | -1.4, -0.31 | 0.002 |
| PGY2 | -0.18 | -0.61, 0.26 | 0.4 |
| Abbreviation: CI = Confidence Interval | | | |

## STROBE 17: Other analyses

Do residents tend to stick in their tertile, move up, or move down?

```
library(ggalluvial)
gg_fig_alluvial <- with_tertiles |>
    group_by(tertile_intern, tertile_pgy2, tertile_senior) |>
    summarise(
        n = n(),
        .groups = 'keep'
    ) |>
    tidyr::complete() |>
    ungroup() |>
    mutate(across(starts_with('t'), \(x) {
        factor(case_match(x,
            1 ~ 'Lowest',
            2 ~ 'Middle',
            3 ~ 'Highest'
        ), levels = c('Lowest', 'Middle', 'Highest'))
    })) |>
    ggplot(aes(
        y = n,
        axis1 = forcats::fct_rev(tertile_intern),
        axis2 = forcats::fct_rev(tertile_pgy2),
        axis3 = forcats::fct_rev(tertile_senior)
    )) +
    geom_alluvium(aes(fill = tertile_intern), width = 1/12) +
    geom_stratum(width = 1/12, fill = "black", color = "grey") +
    geom_label(stat = "stratum", aes(label = after_stat(stratum)), angle = 90) +
    scale_x_continuous(
        name = 'Residency Year',
        breaks = c(1:3),
        labels = \(x) {glue::glue("PGY-{x}")},
        expand = expansion(0)
    ) +
    scale_y_continuous(
        name = 'Tertiles of PGY-1 Residents',
        breaks = NULL,
        expand = expansion(0)
    ) +
    scale_fill_manual(
        name = 'PGY-1 Tertile',
        values = c(
            'Lowest' = 'darkred',
            'Middle' = 'darkblue',
            'Highest' = 'darkgreen'
        )
    ) +
    labs(
        caption = 'Thickness of ribbon indicates number of Residents'
    ) +
    theme_bc_paper()
gg_fig_alluvial
```

Figure 2: Alluvial Plot

### Top or Bottom All the way through

However, X% of residents did stay in the top tertile for all three PGYs, and X% of residents stayed in the bottom tertile for all 3 PGYs. This is [more/less] than would be expected by chance.

```
n_all_first_tertile = nrow(filter(with_tertiles, tertile_intern == 1, tertile_pgy2 == 1, tertile_senior == 1))
n_all_last_tertile  = nrow(filter(with_tertiles, tertile_intern == 3, tertile_pgy2 == 3, tertile_senior == 3))

# Statistical Tests
p_all_first_tertile = n_all_first_tertile / res$n_residents
p_all_last_tertile = n_all_last_tertile / res$n_residents
p_expected = 3 ^ -3

prop.test(
    x = p_all_first_tertile,
    n = res$n_residents,
    p = p_expected
)

    1-sample proportions test with continuity correction

data:  p_all_first_tertile out of res$n_residents, null probability p_expected
X-squared = 2.5758, df = 1, p-value = 0.1085
alternative hypothesis: true p is not equal to 0.03703704
95 percent confidence interval:
 0.0000000 0.0498318
sample estimates:
           p
0.0002312406
prop.test(
    x = p_all_last_tertile,
    n = res$n_residents,
    p = p_expected
)

    1-sample proportions test with continuity correction

data:  p_all_last_tertile out of res$n_residents, null probability p_expected
X-squared = 2.5569, df = 1, p-value = 0.1098
alternative hypothesis: true p is not equal to 0.03703704
95 percent confidence interval:
 0.00000000 0.05003114
sample estimates:
           p
0.0003468609
```

However, 2 / 93 (2.15%) of residents did stay in the top tertile for all three PGYs, and 3 / 93 (3.23%) of residents stayed in the bottom tertile for all 3 PGYs. This is less than would be expected by chance, which suggests at least some level of intention by residents to increase or not focus on increasing the number of critically ill patients they treat.

### Sensitivity analysis: COVID

Table 6

x_by_resident <- bc_by_resident_by_month |>
 group_by(
 pgy = case_match(r,
 c(1:12) ~ 'Intern',
 c(13:24) ~ 'PGY2',
 c(25:37) ~ 'Senior'
 ),
 resident,
 future_critcare_fellow
 ) |>
 filter(!(year(max(month)) %in% c(2020:2021))) |>
 summarise(
 all_pts_this_year = sum(all_patients),
 all_bc_pts_this_year = sum(blackcloud_patients),
 cummulative_bc_pats = max(cumulative_bcs),
 .groups = 'drop'
 )

x_with_tertiles <- x_by_resident |>
 group_by(pgy) |>
 mutate(
 n_residents = n(),
 rank = row_number(all_bc_pts_this_year),
 tertile = ntile(all_bc_pts_this_year, 3)
 ) |>
 pivot_wider(
 id_cols = resident,
 names_from = pgy,
 names_prefix = 'tertile',
 names_sep = '_',
 values_from = tertile
 ) |>
 janitor::clean_names() |>
 tidyr::drop_na()

```
table(x_with_tertiles$tertile_intern, x_with_tertiles$tertile_senior) |>
    chisq.test()

    Pearson's Chi-squared test

data:  table(x_with_tertiles$tertile_intern, x_with_tertiles$tertile_senior)
X-squared = 12.674, df = 4, p-value = 0.01298
table(x_with_tertiles$tertile_intern, x_with_tertiles$tertile_senior) |>
    psych::wkappa()
$kappa
[1] -0.3037889

$weighted.kappa
[1] -0.3797064
table(x_with_tertiles$tertile_intern, x_with_tertiles$tertile_senior) |>
    psych::cohen.kappa(n.obs = nrow(x_with_tertiles))
Call: cohen.kappa1(x = x, w = w, n.obs = n.obs, alpha = alpha, levels = levels,
    w.exp = w.exp)

Cohen Kappa and Weighted Kappa correlation coefficients and confidence boundaries
                 lower estimate upper
unweighted kappa -0.45    -0.30 -0.16
weighted kappa   -0.60    -0.38 -0.16

 Number of subjects = 47
x_with_tertiles  |>
    labelled::set_variable_labels(
        tertile_intern = 'PGY-1 Tertile',
        tertile_senior = 'PGY-3 Tertile'
    ) |>
    gtsummary::tbl_cross(
        row = tertile_intern,
        col = tertile_senior
    )
```

Table 7: Concordance of being in the top tertile across residency (Without)

|  | PGY-3 Tertile | | |  |
| --- | --- | --- | --- | --- |
|  | 1 | 2 | 3 | Total |
| PGY-1 Tertile |  |  |  |  |
| 1 | 3 | 8 | 5 | 16 |
| 2 | 5 | 2 | 6 | 13 |
| 3 | 12 | 5 | 1 | 18 |
| Total | 20 | 15 | 12 | 47 |

Funadmentally unchanged.

### Sensitivity: Adjusting for total patients seen.

```
lin_model_with_total <- lm(
    formula = 'all_bc_pts_this_year_Senior ~ all_bc_pts_this_year_Intern +
                        all_bc_pts_this_year_PGY2 +
                        all_pts_this_year_Senior
    ',
    data = x_by_resident |>
        tidyr::pivot_wider(
            id_cols = resident,
            names_from = pgy,
            values_from = c(
                all_bc_pts_this_year,
                all_pts_this_year
            )
        )
)

gtsummary::tbl_regression(
    lin_model_with_total,
    intercept = TRUE,
    label = list(
        all_bc_pts_this_year_Senior = 'Acutely Ill Patients Seen as PGY-3 Resident',
        all_bc_pts_this_year_Intern = 'Acutely Ill Patients Seen as PGY-1',
        all_bc_pts_this_year_PGY2  = 'Acutely Ill Patients Seen as PGY-2 Resident',
        all_pts_this_year_Senior = 'Total Patients Seen as PGY-3 Resident'
    )
)
```

Table 8: Linear Model Adjusted for Total Volumes

| **Characteristic** | **Beta** | **95% CI** | **p-value** |
| --- | --- | --- | --- |
| (Intercept) | 33 | -37, 104 | 0.3 |
| Acutely Ill Patients Seen as PGY-1 | -0.80 | -1.5, -0.13 | 0.020 |
| Acutely Ill Patients Seen as PGY-2 Resident | 0.15 | -0.43, 0.73 | 0.6 |
| Total Patients Seen as PGY-3 Resident | 0.05 | 0.01, 0.09 | 0.014 |
| Abbreviation: CI = Confidence Interval | | | |

# Discussion

## STROBE 18: Key results summary

Among all residents, there was actually a negative association between being in the top (3rd) tertile for treating acutely ill patients as a PGY-1 with being the top tertile as a PGY-3 resident. This was true for residents in general as well as those who went on to do a fellowship in critical care and resuscitation medicine. Figure 2 shows that there is actually a great deal of movement between tertiles across years.

## STROBE 19: Limitations

- This is a single academic medical center with a three-year residency. While there is no reason to think that a four-year residency would have substantively different results, the culture and catchment area of this center is not representative of other residency programs and patient populations.
- Some acutely ill EHR phenotypes, especially patients triaged as ESI 1, are clearly indicated in the EHR at the time the resident signs up for the patient. Patients identified as acutely ill by diagnosis could not, strictly speaking, be known as qualifying for our criteria at the time the resident signed up to treat the patient. However, we assume the resident generally knows whether he or she is signing up for an acutely ill patient.
- This analysis does not assess quality of care
- This analysis does not control for the possible confounder of rotation order. E.g. is it possible that residents who do their anesthesia rotation earlier during PGY-1 see more critically ill patients? Another analysis we are working on will address this question.

## STROBE 20: Interpretation

We hypothesize that there may be a tendency that once a resident thinks they are ahead of the pack and that perception is reinforced by peers and faculty, there is less urgency to maintain that perception. Conversely, residents may self-identify deficiencies by seeing critically ill patients and actively seek out these encounters independently. Self-assessment theory.

## STROBE 21: Generalizability

Although the specific rates are unlikely to generalize outside this health system with its unique patient population and practice patterns, the absence of a positive association between acutely ill patients seen PGY-1 and PGY-3 year of residency is much more likely to be seen in other contexts, though further research is needed to affirm the presence of a negative association between acutely ill patients seen as an PGY-1 and those seen as a PGY-3 resident.

## STROBE 22 Funding source and role

This work did not receive any external funding and was undertaken as part of a quality assurance initiative. None of the authors have any conflicts of interest to disclose.

# Save images

ggsave('gg_fig_alluvial.PNG', gg_fig_alluvial)
ggsave('gg_fig_trajectories.PNG', gg_fig_trajectories)
